# Cardiovascular Effects of Testosterone Replacement Therapy in Hypogonadal Men: A Systematic Review of Lipid Profiles, Inflammatory Markers, and Vascular Function

**DOI:** 10.7759/cureus.99456

**Published:** 2025-12-17

**Authors:** Patra C Ezeamii, Afolake A Adebayo, Kingsley O Ozojide, Theophilus Kutin Siaw, Kuukua K Ghartey, Ifiok Umana, Chukwujindu I Arinzechi, Edidiong Enyeneokpon, Feyisayo O Oguntuase, Okelue E Okobi

**Affiliations:** 1 Epidemiology and Public Health, Jiann-Ping Hsu College of Public Health, Georgia Southern University, Statesboro, USA; 2 Family Medicine, Nnamdi Azikiwe University, Nnewi, NGA; 3 Public Health, Nottingham Trent University, Nottingham, GBR; 4 Accident and Emergency, Korle Bu Teaching Hospital, Accra, GHA; 5 Urology, Jos University Teaching Hospital, Jos, NGA; 6 Internal Medicine, Savanna-La-Mar Public General Hospital, Savanna-La-Mar, JAM; 7 Acute Medicine, Pilgrim Hospital Boston, Boston, GBR; 8 General Medicine, National Pirogov Memorial Medical University, Vinnytsia, UKR; 9 Family Medicine, Larkin Community Hospital Palm Springs Campus, Hialeah, USA; 10 Family Medicine, IMG Research Academy and Consulting LLC, Homestead, USA

**Keywords:** cardiovascular risk, hypogonadism, inflammation, lipid profile, systematic review, testosterone replacement therapy, vascular function

## Abstract

Testosterone replacement therapy (TRT) is commonly used to treat men with hypogonadism in order to replace the physiological levels of testosterone, although the cardiovascular safety and metabolic advantages of this treatment are still controversial. This article is a systematic review of the existing literature on the cardiovascular impact of TRT on lipid levels, inflammatory parameters, as well as endothelial activity in hypogonadal men. This systematic review was conducted following the Preferred Reporting Items for Systematic Reviews and Meta-Analyses (PRISMA) guidelines. Search was conducted in PubMed, Scopus, Embase, Web of Science, and Google Scholar of peer-reviewed studies that were published between 2005 and 2025. The Revised Assessment of Multiple Systematic Reviews (R-AMSTAR) tool was used to evaluate methodological quality, and the data were synthesized narratively due to substantial heterogeneity across study designs, including differences in TRT formulations, dosing regimens, treatment duration, and the clinical characteristics of hypogonadal populations. Quality ratings were considered when interpreting the evidence, with higher-quality studies providing stronger support for the cardiovascular effects of TRT. In this review, 25 studies were included as they met the inclusion criteria. The included studies comprised randomized controlled trials and observational research designs (systematic reviews, meta-analyses, and cohort studies). Overall, TRT was associated with reductions in total cholesterol (TC) and low-density lipoprotein cholesterol (LDL-C), while high-density lipoprotein cholesterol (HDL-C) showed modest increases or remained stable across studies. Anti-inflammatory effects of testosterone therapy were demonstrated by the decrease in pro-inflammatory cytokines (interleukin-6 (IL-6), tumor necrosis factor-α (TNF-α)) and the enhancement of endothelial-dependent vasodilation.

In hypogonadal men, TRT shows some improvements in endothelial function and reduced systemic inflammation, with benefits most evident under physiological dosing, while its lipid effects may be generally neutral to mildly favorable (decrease in TC/LDL, HDL often somewhat decreased depending on formulation). Long-term cardiovascular safety appears generally reassuring with respect to major adverse cardiac events in appropriately selected patients, though some adverse events may warrant monitoring. High-quality, longer-duration trials remain needed to strengthen these conclusions.

## Introduction and background

Testosterone is a vital hormone that affects the body of males, including sexual activity, mood, body mass, bone strength, and general metabolic conditions [1]. Hypogonadism is a clinical disorder, which is marked by low serum testosterone and is manifested in a considerable percentage of the male population, especially as they age [2]. Prevalence estimates vary, as many reports include isolated biochemical low testosterone, which may be present in up to 20% of men over 60 and does not always indicate symptomatic hypogonadism [3]. The effects of hypogonadism diffusion tend to make the patients require testosterone replacement therapy (TRT) to restore the hormonal equilibrium and the quality of life [4,5].

In the last 20 years, there has been a significant rise in the utilization of TRT due to a rise in clinical awareness, direct-to-consumer marketing, and an expansion in diagnostic testing, but it remains unclear how much of this growth reflects appropriately diagnosed symptomatic hypogonadism versus potential overdiagnosis, a distinction that is important when considering the overall risk-benefit profile of TRT [6]. The cardiovascular safety of TRT is, however, a subject of debate and clinical ambiguity [7]. Although testosterone has a positive impact on body composition and insulin sensitivity, it can also affect lipid metabolism as well as vascular tone and inflammatory pathways, which are important determinants of cardiovascular health [8,9]. There was an early indication of the risk, such as augmented myocardial infarction and stroke in TRT users by observational and review studies [10]. However, the systematic reviews have suggested a neutral or even protective effect on the cardiovascular systems of hypogonadal men using appropriate therapy [11,12].

Among the suggested pathways through which testosterone influences cardiovascular events is by virtue of altering the lipid profiles [13]. A number of studies have reported a TRT-related decrease in total cholesterol (TC), low-density lipoprotein cholesterol (LDL-C), but its impact on high-density lipoprotein cholesterol (HDL-C) is variable [14]. Besides that, testosterone can also affect systemic inflammation, which can be assessed with C-reactive protein (CRP) and interleukin-6 (IL-6), which may contribute to atherosclerotic risk. However, the extent to which changes in these biomarkers translate into measurable cardiovascular outcomes remains uncertain [15,16]. In addition, the effects of testosterone on vascular function, such as endothelial reactivity, arterial stiffness, and nitric oxide (NO) bioavailability, have also been acknowledged as an important bridge between hormonal status and cardiovascular health. However, these vascular effects have not been demonstrated consistently across human clinical studies, with findings varying by study design and TRT duration [17].

Although research has increased, results are diverse, and variations in the study design, TRT formulations, dosing schedules, and population attributes are involved in the variation of reported results. It is thus necessary to conduct a systematic review to integrate the existing evidence and explain the cardiovascular consequences of TRT in hypogonadal men. The objective of the review is to determine the effect of testosterone replacement on lipid levels, inflammatory biomarkers, and vascular health, which will give a holistic picture of the possible cardiovascular hazards and advantages. While the primary focus is on these intermediate outcomes, relevant evidence on clinical cardiovascular events reported within included studies is also considered where available.

## Review

Methods

Eligibility Criteria and Search Strategies

To maintain transparency, methodology rigor, and reproducibility throughout the process of research, this systematic review was carried out as per the Preferred Reporting Items for Systematic Reviews and Meta-Analyses (PRISMA) 2020 guidelines [18]. The study utilized the Population, Intervention, Comparison, and Outcome (PICO) framework in order to determine the search and selection strategy: Population (adult males diagnosed with hypogonadism), Intervention (TRT in any form injectable, oral, transdermal, or implantable), Comparison (placebo, no therapy, or delayed therapy), and Outcomes (cardiovascular parameters such as lipid profiles, inflammatory markers, and vascular/endothelial activity).

The literature review was limited to peer-reviewed articles published in English from January 2005 to July 2025. This restriction may introduce language bias, and we acknowledge that relevant non-English studies may not have been captured.

The search was performed in five electronic databases with PubMed, Scopus, Web of Science, and Embase. Such databases were chosen because they are very broad in terms of coverage of biomedical and clinical research. Relevant keywords were combined using Boolean operators, Medical Subject Headings (MeSH), and as presented in Table 1.

**Table 1:** Keywords used for the literature search

Category	Details
Databases Searched	PubMed, Scopus, Web of Science, Embase
Time Frame	January 2005-July 2025
Language	English only
Search Terms	#1 AND #2 AND #3
#1 (Population)	“Hypogonadal men” OR “male hypogonadism” OR “low testosterone”
#2 (Intervention)	“Testosterone replacement therapy” OR “testosterone supplementation” OR “androgen therapy” OR “TRT”
#3 (Outcomes)	“Cardiovascular effects” OR “lipid profile” OR “inflammatory markers” OR “vascular function” OR “endothelial function” OR “C-reactive protein” OR “cholesterol”

Inclusion Criteria

The inclusion criteria included that the studies had to be of interest, looking at the relationship between TRT and cardiovascular outcomes in a group of hypogonadal men. Qualifiable studies were systematic reviews that used lipid profiles or inflammatory markers, or measures of vascular function (e.g., flow-mediated dilation (FMD)). Systematic reviews were included for contextual understanding but were synthesized separately from primary studies to avoid duplication of evidence and prevent overweighing of findings in the narrative synthesis. Original peer-reviewed articles published in English were included to maintain methodological consistency and avoid potential translation errors that could affect data accuracy and comparability across studies.

Exclusion Criteria

To ensure the integrity and relevance of data, exclusion criteria were used. Articles that researched TRT in women or transgender, animal, or in vitro research, editorials, letters to the editor, dissertations, and conference abstracts were eliminated. Studies that did not report any cardiovascular parameters were excluded. For mixed-outcome studies in which cardiovascular measures were secondary endpoints, inclusion was permitted as long as the cardiovascular data were clearly reported and met the review’s outcome criteria.

Screening Process

All identified studies were imported to EndNote reference manager (Clarivate, London, UK) in order to eliminate duplicates. Titles and abstracts were screened by two reviewers who were independent and were used to select relevant articles in accordance with predetermined inclusion criteria. The differences between reviewers were eliminated by discussing, and in case of disagreement, a third reviewer gave a final decision. To record the selection process, a PRISMA flow diagram was created to demonstrate the number of records that had been identified, screened, and included or excluded at each step.

Quality Assessment

In assessing the quality of the articles, suitable instruments were used based on the study design. The Revised Assessment of Multiple Systematic Reviews (R-AMSTAR) tool was applied to assess the selected articles [19].

Data Extraction

The collection of the key information was designed in the form of a standardized data extraction form. The information extracted was authors, year of publication, country, study design, sample size, age range, testosterone dose and route, duration of therapy, cardiovascular effects (lipid levels, inflammatory markers, endothelial function), and key findings. To eliminate the risk of bias in data extraction, two reviewers extracted data independently, and differences were resolved by discussion.

Data Analysis

Data analysis was conducted using a narrative synthesis approach due to substantial heterogeneity across the included studies, particularly in TRT formulations, dosing regimens, outcome definitions, and measurement time points. These variations limited the feasibility of performing a formal meta-analysis. To organize the findings, studies were grouped into three thematic domains: lipid metabolism, inflammatory and metabolic biomarkers, and vascular and endothelial function. Within each domain, results were summarized descriptively to highlight patterns, inconsistencies, and mechanistic insights relevant to cardiovascular health in hypogonadal men. This qualitative synthesis allowed for an integrated interpretation of outcomes while accommodating the methodological diversity of the included research.

Results

Firstly, the search of the database had 580 studies, and 102 of them were duplicates. Following a screening of 478 titles and abstracts, 400 were eliminated because of irrelevance or a lack of inclusion in the study. The rest of the full-text articles were evaluated as eligible, and 53 were eliminated due to the insufficiency of cardiovascular information, lack of human focus, or non-peer review. Finally, 25 studies were included in the systematic review as they met the inclusion criteria.

Geographically, the studies were of a broad scope comprising North America, Europe, and Asia, with the majority of the studies being done in the clinical setting, with the majority of the men aged between 40 and 75 years. The featured studies were systematic reviews, which provided a variety of research methodologies.

**Figure 1:**
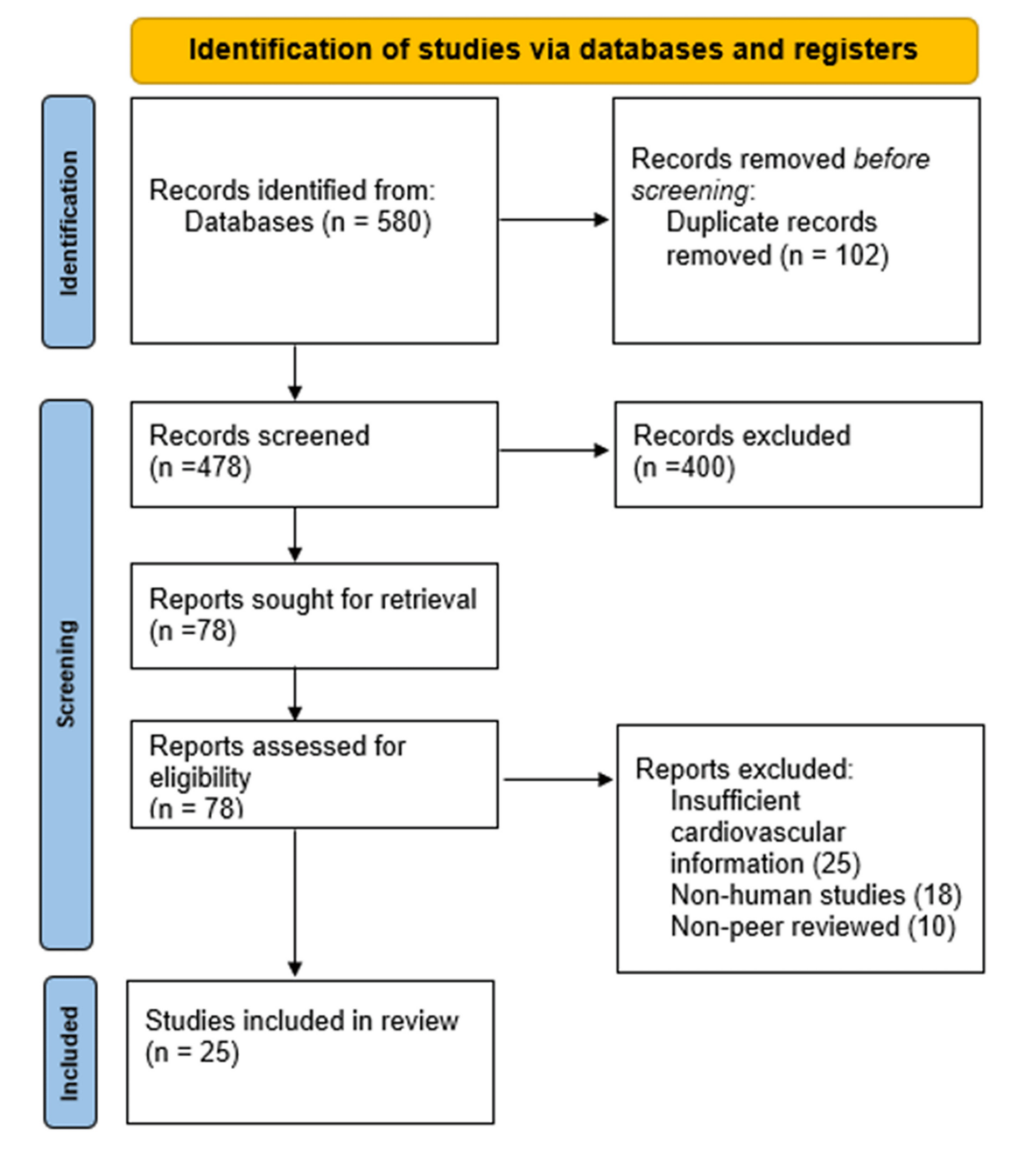
PRISMA flow diagram indicating the study selection and inclusion process PRISMA: Preferred Reporting Items for Systematic Reviews and Meta-Analyses

Figure 1 presents the PRISMA flow diagram demonstrating the study selection and inclusion process of the required studies.

Table 2 presents the summary characteristics of the 25 studies included in the review, including study design, population, outcome focus, and key findings.

**Table 2:** Summary of included studies TRT: testosterone replacement therapy; T2DM: type 2 diabetes mellitus; DHT: dihydrotestosterone; LDL: low-density lipoprotein; HDL: high-density lipoprotein; CRP: C-reactive protein; IL-6: interlukin-6

Reference	Study Design	Population	Outcome Focus	Key Findings
Elliott et al. (2017) [1]	Systematic review and network meta-analysis	Hypogonadal men	Lipid profile, cardiovascular outcomes	TRT improved lipid metabolism and sexual function without a significant increase in cardiovascular risk.
Millar et al. (2016) [2]	Systematic review	Aging men	Testosterone prediction and deficiency screening	Identified predictors for hypogonadism; emphasized metabolic and lipid implications.
Thirumalai and Anawalt (2022) [3]	Epidemiological review	Global male population	Prevalence of hypogonadism	Highlighted the increasing prevalence linked with metabolic syndrome and aging.
Guo et al. (2016) [4]	Meta-analysis review	Men with hypogonadism	Safety and efficacy of TRT	TRT improved lipid levels and energy but required monitoring for cardiovascular risk.
Xu et al. (2024) [5]	Systematic review and meta-analysis	Hypogonadal men	Erectile and prostate function	TRT improved erectile function; no rise in cardiovascular or prostate risks.
Bandari et al. (2017) [6]	Systematic review	U.S. population	Testosterone marketing	Discussed overprescription and emphasized the need for evidence-based therapy.
Xu et al. (2013) [7]	Systematic review and meta-analysis	Men under TRT	Cardiovascular events	No clear evidence of increased cardiovascular risk with physiological TRT.
Li et al. (2020) [8]	Meta-analysis review	Men with T2DM/metabolic syndrome	Metabolic outcomes	TRT improved insulin sensitivity and reduced LDL cholesterol.
Mlynarz et al. (2024) [9]	Systematic review	Men with metabolic syndrome	Metabolic profile	TRT reduced waist circumference and improved lipid parameters.
Bianchi (2018) [10]	Review	Men under TRT	Inflammatory markers	Testosterone exhibited anti-inflammatory effects by reducing CRP and IL-6.
Jones and Kelly (2018) [11]	Systematic review	Hypogonadal men	Cardiovascular mechanisms	TRT improved endothelial function and vascular reactivity.
Sansone et al. (2020) [12]	Systematic review and meta-analysis	Hypogonadal men	Endothelial function	TRT improved flow-mediated dilation and reduced vascular stiffness.
Lee et al. (2024) [13]	Cochrane systematic review	Men with sexual dysfunction	Sexual and cardiovascular safety	TRT improved sexual function without increasing adverse events.
Huo et al. (2016) [14]	Systematic review	Men with low testosterone	Treatment patterns	Found variable cardiovascular reporting; called for standardized safety endpoints.
Traish et al. (2018) [15]	Review	Hypogonadal men	Inflammation and vascular pathways	Demonstrated androgen modulation of cytokine and nitric oxide pathways.
Mohamad et al. (2019) [16]	Systematic review	Aging men	Inflammatory cytokines	Inverse correlation between testosterone levels and pro-inflammatory markers.
Cai et al. (2014) [17]	Systematic review and meta-analysis	Hypogonadal men with T2DM	Metabolic and lipid profile	TRT improved HDL and reduced triglycerides and insulin resistance.
Zarotsky et al. (2014) [20]	Systematic review	Global male cohort	Risk factors and comorbidities	Identified cardiovascular and metabolic risk factors in hypogonadal men.
Cruickshank et al. (2024) [21]	Evidence synthesis and economic evaluation review	Men with hypogonadism	Safety and cost-effectiveness	Found TRT clinically beneficial and cost-effective when properly monitored.
Zhou et al. (2020) [22]	Systematic review	U.S. Medicare data	TRT trends	Documented increased TRT use with improved cardiovascular monitoring.
Borst et al. (2014) [23]	Systematic review and meta-analysis	Hypogonadal men	DHT levels and cardiovascular risk	Route of administration affected DHT and lipid levels; transdermal is safer.
Isidori et al. (2005) [24]	Meta-analysis review	Middle-aged men	Body composition and lipid profile	TRT reduced fat mass and total cholesterol, and improved HDL.
Kim et al. (2021) [25]	Systematic review and meta-analysis	Men with late-onset hypogonadism	Metabolic disturbances	TRT improved glycemic control and lipid regulation.
Hotta et al. (2019) [26]	Systematic review	Men with testosterone deficiency	Endothelial dysfunction	Low testosterone is linked to increased oxidative stress and vascular stiffness.
Lopes et al. (2012) [27]	Review	Aging men	Vascular physiology	Testosterone maintained endothelial integrity and vascular tone in aging males.

Table 3 reports the quality assessment of the included systematic reviews based on the R-AMSTAR evaluation framework, which assigns scores across 11 methodological criteria. R-AMSTAR scores range from 0 to 11; scores ≥8 were interpreted as high quality, 5-7 as moderate quality, and ≤4 as low quality.

**Table 3:** Assessment of the included systematic reviews using the R-AMSTAR tool Item 1: a priori design; item 2: duplicate study selection and data extraction; item 3: comprehensive literature search; item 4: publication status as an inclusion criteria; item 5: list of included and excluded studies; item 6: characteristics of included studies; item 7: documented assessment of the scientific quality of included studies; item 8: appropriate use of the scientific quality in forming conclusions; item 9: appropriate use of methods to combine study findings; item 10: assessment of publication bias likelihood; item 11: conflict of interest documentation. R-AMSTAR: Revised Assessment of Multiple Systematic Reviews Source: Reference [19]

Study (Year)	1	2	3	4	5	6	7	8	9	10	11	Score
Elliott et al. (2017) [1]	1	1	1	1	1	1	1	1	1	1	1	11
Millar et al. (2016) [2]	1	1	1	1	1	1	1	0	1	0	1	9
Thirumalai and Anawalt (2022) [3]	1	1	1	0	1	1	1	0	1	0	1	8
Guo et al. (2016) [4]	1	1	1	1	1	1	1	1	1	1	0	10
Xu et al. (2024) [5]	1	1	1	1	1	1	1	1	1	1	1	11
Bandari et al. (2017) [6]	1	1	1	0	1	1	1	0	1	0	0	7
Xu et al. (2013) [7]	1	1	1	1	1	1	1	1	1	1	0	10
Li et al. (2020) [8]	1	1	1	1	1	1	1	0	1	0	1	9
Mlynarz et al. (2024) [9]	1	1	1	1	1	1	1	1	1	0	1	10
Bianchi (2018) [10]	1	1	1	0	1	1	1	0	1	0	0	7
Jones and Kelly (2018) [11]	1	1	1	0	1	1	1	0	1	1	0	8
Sansone et al. (2020) [12]	1	1	1	1	1	1	1	1	1	1	1	11
Lee et al. (2024) [13]	1	1	1	1	1	1	1	0	1	0	1	9
Huo et al. (2016) [14]	1	1	1	1	1	1	1	0	1	0	1	9
Traish et al. (2018) [15]	1	1	1	0	1	1	1	0	1	0	1	8
Mohamad et al. (2019) [16]	1	1	1	0	1	1	1	0	1	0	1	8
Cai et al. (2014) [17]	1	1	1	1	1	1	1	0	1	0	1	9
Zarotsky et al. (2014) [20]	1	1	1	1	1	1	1	1	1	1	1	11
Cruickshank et al. (2024) [21]	1	1	1	1	1	1	1	0	1	0	1	9
Zhou et al. (2020) [22]	1	1	1	1	1	1	1	0	1	1	1	10
Borst et al. (2014) [23]	1	1	1	1	1	1	1	1	1	0	1	10
Isidori et al. (2005) [24]	1	1	1	0	1	1	1	0	1	0	0	7
Kim et al. (2021) [25]	1	1	1	0	1	1	1	0	1	0	1	8
Hotta et al. (2019) [26]	1	1	1	1	1	1	1	1	1	1	1	11
Lopes et al. (2012) [27]	1	1	1	0	1	1	1	0	1	0	1	8

Study findings

This systematic review synthesized 25 studies of the cardiovascular effects of TRT in hypogonadal men in terms of lipid profiles, inflammatory markers, and vascular functionality [20,21]. The findings from these articles support the idea that TRT has positive and neutral cardiovascular effects that are conditional on the factors of patient features, duration of treatment, and the method of its intake [22,23].

Three consistent findings were identified through thematic synthesis. To begin with, TRT positively affected lipid metabolism with considerable efficiency by reducing total and LDL cholesterol and modestly elevating HDL cholesterol. Second, anti-inflammatory effects were evidenced in several studies, with reductions in CRP and pro-inflammatory cytokines like IL-6 and tumor necrosis factor-α (TNF-α), proclaiming the cardiovascular protective properties of testosterone. Third, TRT showed positive effects on endothelial and vascular activities, increasing the bioavailability of NO and FMD, which is a consequence of vascular health.

Nonetheless, some of the studies expressed some concerns over the possibility of cardiovascular risks, especially with supraphysiologic dosage or even with sustained treatment. In general, the results of this systematic review indicate that physiological testosterone replacement in hypogonadal men is likely to have a beneficial effect on cardiovascular biomarkers but does not lead to more adverse cardiac events when adequately monitored.

One of the major similarities observed in the literature was the positive lipid modulation after TRT. The majority of the trials reported a decrease in total and LDL cholesterol, and an increase or preservation of HDL cholesterol in men with hypogonadal population under treatment [24]. Such lipid alterations have clinical significance in that they are indicative of cardiovascular risk factor improvements. Besides, meta-analytical evidence indicated that long-term TRT also represents the occurrence of a decrease in triglyceride levels and general metabolic risk, especially in male patients with late-onset hypogonadism [25].

In addition to the lipid control, TRT showed anti-inflammatory properties, which were evidenced by the decreasing levels of pro-inflammatory cytokines in the circulation, including TNF-α, IL-6, and CRP [21]. Such positive effects on inflammatory response profiles indicate that testosterone is a modulatory factor in immune-endocrine responses that can help prevent atherosclerotic progression. It was shown that with restoration of normal levels of testosterone in hypogonadal men, oxidative stress and endothelial dysfunction are minimized and are contributors to vascular protection [26]. The anti-inflammatory, endothelial effects were especially observed in the studies in which TRT has attained physiological testosterone levels without overdosing [27].

In terms of vascular activity, TRT was proven to increase endothelial-dependent vasodilation by NO activation and inhibition of asymmetric dimethylarginine (ADMA), which is a natural inhibitor of NO synthase [26]. Experimental and clinical research proved a positive effect of sustained TRT use on arterial stiffness and FMD, which led to better vascular reactivity and the general condition of cardiovascular health [27]. These effects, however, were most evident among men who had baseline hypogonadism, and non-consistent benefits were observed in the eugonadal subjects who used off-label testosterone [22].

Although these positive results were attained, there are still some variability and safety issues. Some of the studies stated possible risks associated with the type of formulation and route of administration, especially the intramuscular testosterone, which temporarily raised the dihydrotestosterone (DHT) and hematocrit levels, thus putting them at risk of thromboembolism [23]. However, major adverse cardiovascular events (MACEs) have not been shown to significantly rise in large-scale systematic reviews and economic analyses when appropriately monitored and titrated [21].

Overall, this review finds that TRT in hypogonadal men tends to improve lipid metabolism and inflammation and improve vascular performance, and there is no compelling evidence that cardiovascular risk is elevated [22]. Nevertheless, the treatment must be tailored based on the initial cardiovascular condition and observed closely to strike between the advantage and the possible hematologic and vascular adverse effects [20,21,23,27].

Discussion

Effects of TRT on Lipid Profiles

TRT has demonstrated great lipid metabolism effects in hypogonadal men. There is an indication that TRT has a tendency to decrease the TC and LDL and at least slightly decrease the amount of HDL, although the clinical significance of this effect is controversial [8,24]. One meta-analysis has shown that long-term TRT enhances lipid homeostasis by reducing levels of triglycerides and increasing insulin sensitivity in men with metabolic syndrome [17,25]. These results show a positive change in cardiovascular risk factors, especially in diabetic or obese patients. The amount of such benefits, however, tends to be related to the route and dosage used to deliver testosterone, with injectable delivery displaying greater lipid benefits than oral or transdermal delivery routes [23]. TRT has moderate lipid-modulating effects, which improve cardiovascular profiles, but which will only be proven significant in large populations through subsequent large-scale research studies [4].

Anti-inflammatory and Immunomodulatory Effects of Testosterone Therapy

Testosterone possesses significant anti-inflammatory effects, which affect a variety of immune pathways and cytokine profiles. Research shows that TRT decreases systemic inflammation through decreasing pro-inflammatory cytokines like CRP, TNF-α, and IL-6 [10,15,16]. The macrophage and endothelial cell modulatory effects of the hormone enhance vascular repair and minimize oxidative stress, which facilitates cardio protection [11,27]. Mechanistic hypotheses postulate that testosterone triggers androgen receptors, which in turn inhibit the nuclear factor-kappa B (NF-kB) pathway that inhibits inflammatory signaling and atherosclerotic plaque development [10]. Also, TRT rejuvenates endothelial progenitor cells, which facilitates vascular recovery and endurance [26]. Although it is indicated that testosterone has anti-inflammatory effects, the excessive or supra-physiologic dosing can enhance risks associated with erythrocytosis and viscosity [21]. Thus, it is essential to keep the physiological levels of testosterone in order to maximize its immunomodulatory and cardiovascular effects without enhancing the negative effects [15,16].

Impact of Testosterone Therapy on Endothelial and Vascular Function

TRT has been repeatedly associated with enhanced endothelial activity and vascular reactivity in hypogonadal men. Testosterone increases the production of NO, which increases vasodilation and arterial compliance [26,27]. According to meta-analyses, treatment has a considerable positive effect on FMD as well as a negative effect on ADMA, a good inhibitor of NO bioavailability [12]. These vascular effects are converted to enhanced coronary perfusion and decreased arterial stiffness, which minimizes cardiovascular risk [11]. Besides, testosterone aids in the mobilization of endothelial progenitor cells to stimulate vascular regeneration and repair [26]. Nevertheless, the outcomes are different based on initial cardiovascular fitness, and men with underlying heart disease are also ambivalent to TRT [23]. However, in proper dosages, TRT increases vascular tone, endothelial health, and arterial elasticity and therefore highlights its therapeutic role in cardiovascular risk modulation in hypogonadal men [12,26].

Limitations

There are some limitations associated with this review. A search was conducted to include studies published between 2005 and 2025 in English, which may have left out relevant non-English or unpublished data. It did not review grey literature, conference proceedings, or current trials, which might have limited comprehensiveness. Also, differences in testosterone preparations, doses, and periods of administration, and population heterogeneity in the studies, created a limitation in the ability to directly compare studies. Lastly, the vast majority of incorporated studies were conducted in high-income countries, so the findings cannot be generalized and applied to a wide variety of global populations.

## Conclusions

This review shows that TRT leads to a better lipid profile, lower pro-inflammatory cytokine levels, and improved endothelial health, all of which may lead to improved cardiovascular outcomes following the right administration. Individualized dosing, the length of treatment, and the underlying cardiovascular risk also demonstrated this benefit. These improvements may contribute to enhanced cardiovascular health and overall well-being in this population. Further monitoring and long-term trials are necessary to clarify the safety profile and long-term effects of TRT to optimize treatment protocols for individual patients.
